# An Optimized CoBRA Method for the Microfluidic Electrophoresis Detection of Breast Cancer Associated *RASSF1* Methylation

**DOI:** 10.3390/biotech12010007

**Published:** 2023-01-09

**Authors:** Claire Aibel, Adriana Coll De Peña, Anubhav Tripathi

**Affiliations:** 1 Division of Biology and Medicine, Brown University, Providence, RI 02912, USA; 2 Center for Biomedical Engineering, School of Engineering, Brown University, Providence, RI 02912, USA

**Keywords:** microfluidic electrophoresis, combined bisulfite restriction analysis, breast cancer, epigenetics, methylation

## Abstract

Although breast cancer screening assays exist, many are inaccessible and have high turnaround times, leaving a significant need for better alternatives. Hypermethylation of tumor suppressor genes is a common epigenetic marker of breast cancer. Methylation tends to occur most frequently in the promoter and first exon regions of genes. Preliminary screening tests are crucial for informing patients whether they should pursue more involved testing. We selected *RASSF1*, previously demonstrated to be aberrantly methylated in liquid biopsies from breast cancer patients, as our gene of interest. Using CoBRA as our method for methylation quantification, we designed unique primer sets that amplify a portion of the CpG island spanning the 5′ end of the *RASSF1* first exon. We integrated the CoBRA approach with a microfluidics-based electrophoresis quantification system (LabChip) and optimized the assay such that insightful results could be obtained without post-PCR purification or concentration, two steps traditionally included in CoBRA assays. Circumventing these steps resulted in a decreased turnaround time and mitigated the laboratory machinery and reagent requirements. Our streamlined technique has an estimated limit of detection of 9.1 ng/μL of input DNA and was able to quantify methylation with an average error of 4.3%.

## 1. Introduction

Approximately 13% of women in the United States will develop breast cancer during their lifetime, a proportion that increases by about 0.5% each year [1,2]. Some patients go into remission with treatment, but often not without substantial long-term financial, physical, and psychological burdens [3]. It is crucial for symptomatic women—regardless of age—to promptly undergo screening tests, as this can mitigate their risk of developing late-stage breast cancer. In addition, asymptomatic patients who are over 50 years old should undergo systematic screening tests, which can decrease mortality by 20–30% [4,5]. Some findings suggest that recurring asymptomatic screening tests may have similar benefits among people between 40 and 50 years of age [5]. However, these tests are often inaccessible, especially for patients who are socioeconomically disadvantaged, underinsured, or uninsured. 

Mammograms are considered the gold standard for non-invasive early detection, but are generally only covered by insurance after age 40 [6]. Furthermore, the probability of at least one false-positive result when undergoing annual mammograms over a ten-year period is around 50%, which may lead to anxiety and unnecessary tissue biopsies [7,8]. Genetic screening is becoming increasingly popular, but only 5 to 10% of breast cancer cases are due to genetic predisposition [9]. Therefore, screens for mutations in genes such as *BRCA1* and *BRCA2* often come up negative for patients who do have breast cancer, which can create a false sense of security and discourage self-monitoring and future screening.

Because of the drawbacks associated with conventional screening methods, the development of tests that are effective, affordable, timely, and as painless as possible is crucial for the early detection of breast cancer. An emerging focus of breast cancer screening is the analysis of epigenetic modifications, or changes that alter gene expression without the introduction of mutations. DNA methylation is an epigenetic change involving the addition of a methyl group to the C5 position of a cytosine residue [10], which can result in changes in gene expression and carcinogenesis [11]. It has been established that, relative to healthy controls, a substantial number of breast cancer patients display aberrant methylation patterns (Table 1) in several tumor suppressor genes [12]. The promoter and first exon regions of many tumor suppressor genes implicated in breast cancer contain CpG islands, which are regions with a relatively high number of CpG dinucleotides. Abnormal methylation of these regions can cause downregulation or silencing of tumor suppressor genes and result in uncontrolled tumor growth. This association between methylation and breast cancer has motivated a surge of research over the past few decades. Numerous groups have focused on characterizing the prevalence of methylation in various breast cancer-implicated genes (Table 1) to better understand the relationship between methylation and prognosis.

The method by which a patient sample is obtained (i.e., tissue biopsy or liquid biopsy) is important to consider when developing screening assays. To minimize the invasiveness of the protocol, circulating cell-free DNA (ccfDNA) from routine blood draws (i.e., liquid biopsies) was selected as the preferred sample collection method in this study. Thus, the genes of interest for analysis were selected partially based on their methylation incidence in ccfDNA, which can differ from that in tissue biopsies (Table 1). Additionally, it is important to consider the percentage of healthy individuals that also contain the aberrant marker. For instance, when analyzed in ccfDNA, *14-3-3σ* displayed hypermethylation in 65% of cancer patient samples and 38% in non-cancer patient samples [15]. Therefore, despite having one of the highest incidences of methylation in cancerous tissues, *14-3-3σ* also displays methylation in over one-third of healthy patients, making it difficult to use this gene as a screening marker for breast cancer. By contrast, *RASSF1* was methylated in an average of 38% of ccfDNA from cancer patients and only 6% of healthy patient samples [15]. Based on a thorough literature search and preliminary data from analyzing several genes of the highest diagnostic significance from ccfDNA, we decided to focus on *RASSF1* as the target of our screening assay.

The most common method for methylation detection is bisulfite sequencing, a process by which DNA is treated with sodium bisulfite conversion reagents, amplified, and sequenced. During this process, unmethylated cytosines are converted to thymines. Gene sequencing enables the comparison of the number of thymines in the treated DNA with that of the untreated DNA, revealing the methylation status of the gene [33]. However, sequencing of a single gene can range from hundreds to thousands of dollars, and is often costlier than the sequencing of an entire genome [34,35]. Additionally, it can take weeks to receive sequencing results, which can lead to stress and further the progression of the disease [36].

**Figure 1:**
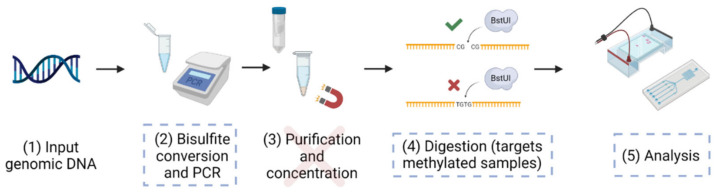
Conventional CoBRA workflow with dashed blue boxes highlighting the areas improved in this study (steps 2, 4, and 5) and a red cross the step that was eliminated (step 3). Traditionally, (1) genomic DNA is (2) treated with bisulfite conversion reagents, which preserve methylated cytosine residues but convert unmethylated cytosines to uracil. Then, the bisulfite-treated DNA undergoes PCR with primers specific to the region of interest. Since PCR utilizes dNTPs, the amplified DNA will ultimately contain thymine residues in place of uracil. PCR products are (3) purified and concentrated. CGCG sequences in the methylated DNA remain as such, whereas those in the unmethylated DNA ultimately become TGTG. Therefore, (4) BstUI will only digest DNA that was originally methylated. (5) Gel electrophoresis or fragment-based microfluidic electrophoresis analysis is used to quantify methylation. Figure created using BioRender.com.

Combined bisulfite restriction analysis (CoBRA) was introduced in 1997 by Xiong and Laird [37]. This technique enables the quantification of methylation in a specific gene without the need for sequencing (Figure 1). In this method, patient DNA is extracted, treated with sodium bisulfite conversion reagents, and amplified with primers that isolate a region from the gene of interest. The PCR products are then digested with an enzyme, generally BstUI, which has a recognition site of CG^CG. In methylated genes, CG sites are retained during the bisulfite-PCR reaction, so BstUI will digest at the preserved CGCG sites. Conversely, in unmethylated genes, CGCG sequences are converted to TGTG during the bisulfite-PCR process, and BstUI will not recognize these altered sites. The resulting digestion products are run on a gel to separate fragments by size. The ratio of intact DNA concentration to digested DNA concentration corresponds to the ratio of unmethylated to methylated DNA in the sample. Imaging software is generally used to estimate the concentration of DNA in each band, but visual interpretation makes accurate quantification challenging and introduces additional variability.

Since Xiong and Laird’s publication, microfluidics has emerged as an alternative method of quantifying DNA fragments with relatively high accuracy. In 2006, Brena et al. published a protocol for “Bio-CoBRA,” a technique that combines the traditional CoBRA assay with a microfluidic electrophoresis-based analysis [38]. While this technique showed significant improvement, our study focuses on developing a system that further streamlines this process (Figure 1).

In this study, we sought to mitigate key drawbacks of current clinical breast cancer screening tests and improve upon existing methylation-detection technologies to develop a streamlined, epigenetics-based approach. First, our primers were designed to isolate part of a CpG island spanning an early region of the *RASSF1* first exon (step 2, Figure 1), which contains two distinct CGCG sites and thus increases the analytical capacity of the system. Next, by taking advantage of recent improvements in enzyme technology, we optimized the CoBRA assay by decreasing digestion time (step 4, Figure 1). Lastly, by using our high sensitivity analytical method (step 5, Figure 1), we bypassed the purification and concentration steps (step 3, Figure 1) generally required in the standard CoBRA [37] and Bio-CoBRA [38] protocols, significantly decreasing turnaround time, apparatus requirements, and reagent use. The BioAnalyzer- and the LabChip-based assays have undergone significant advances in sensitivity and resolution since the study by Brena et al. [38] was published, which we aimed to capitalize upon in this study.

Our high sensitivity and resolution method allowed us to assess methylation percentage by comparing the concentrations of the peak corresponding to *RASSF1* in the presence and absence of the restriction enzyme, instead of the ratio of the digested to the undigested peaks [38] within a given sample. By doing so, our sample analysis did not require the appearance of new peaks—which would have increased the limit of detection (LOD) and sample concentrator apparatus—and enabled us to bypass the need for sample concentration. Similarly, by solely focusing on changes to the primary and intended PCR product, we were able to bypass the need for post-PCR sample purification, as our method does not rely on analysis of the digestion product peaks that would overlap with the amplification byproducts, such as primer dimer. Here, we present an optimized technique that rapidly assesses methylation patterns in *RASSF1* by coupling our streamlined CoBRA protocol with a high throughput, sensitivity, and resolution microfluidics-based electrophoretic system.

## 2. Materials and Methods

### 2.1. Bisulfite Treatment

Fully methylated (M-DNA) and fully unmethylated (UM-DNA) genomic DNA was purchased from Zymo Research, Irvine, CA, USA. The samples of M-DNA and UM-DNA were diluted to 25 ng/μL at a total volume of 20 μL and then treated with bisulfite-conversion reagents from the Zymo EZ DNA Methylation-Lightning Kit following the provided protocol (Zymo Research, Irvine, CA, USA). In the initial experiments to evaluate primer efficacy, M-DNA and UM-DNA were assessed, coupled with a negative control consisting of 20 µL of nuclease-free water (NFW) with no sample DNA. Separate experiments were conducted to evaluate whether the efficacy of the system depended on the initial DNA concentration. For this, the following samples were prepared: 12.5 ng/µL M-DNA, 25 ng/µL M-DNA, and 37.5 ng/µL M-DNA, all at volumes of 20 µL. A negative control, consisting of 2 µL TE buffer + 18 µL NFW, was also prepared to approximate the conductivity of the samples, which is crucial for accuracy in microfluidic electrophoresis. Three additional samples and a negative control, with the same reagent concentrations and volumes, were prepared in parallel using UM-DNA instead of M-DNA.

Next, the quantification accuracy of our system was evaluated by preparing samples with varying percentages of methylation. Three 20 µL samples were prepared with the following percentage methylation: 100% M-DNA (25 ng/µL), 50% M-DNA (10 µL of 12.5 ng/µL M-DNA and 10 µL of 12.5 ng/µL UM-DNA), and 0% M-DNA (25 ng/µL UM-DNA). A negative control, consisting of 2 µL TE buffer + 18 µL NFW, was also prepared. In all experiments, bisulfite reagents were added according to the Zymo EZ DNA Methylation-Lightning Kit protocol. The samples were centrifuged at 15,000 x g for 30 s, and were then incubated at room temperature for 19 min following the addition of the L-Desulphonation Buffer.

### 2.2. Polymerase Chain Reaction (PCR)

The 5′ end of the first exonic region of *RASSF1* was identified using Ensembl, as described by Howe et al. [39]. Integrative Genomics Viewer was used to determine whether the region detected by Ensembl was located within a CpG island following the steps reported in Robinson et al. [40]. This was verified mathematically using the criteria outlined by Gardiner-Garden et al. (1987), who define CpG islands as sequences longer than 200 bp with GC contents of at least 50%, and a value of greater than 0.6 for the following equation [41]:

(1) $$\frac{\mathrm{Observed}\;\mathrm{CpG}}{\mathrm{Expected}\;\mathrm{CpG}}=\frac{\mathrm{Number}\;\mathrm{of}\;\mathrm{CpG}}{\left(\mathrm{Number}\;\mathrm{of}\;C\right)\times \left(\mathrm{Number}\;\mathrm{of}\;G\right)}\times N$$

where N represents the total number of nucleotides (nt) in the sequence of interest. After confirming that the 5′ end of the *RASSF1* first exon was located within a CpG island, PCR primers were designed using the Free Bisulfite Primer Design Tool (Zymo Research, Irvine, CA, USA). The parameters were adjusted such that primers would be ideal for the amplification of bisulfite-treated DNA, with primer lengths between 20 and 36 nt, annealing temperatures between 55 and 60 °C, and amplicon sizes between 150 and 300 nt. Among the amplicons generated by the software, only those containing at least one CGCG site were considered.

To evaluate primer efficacy, 25 µL PCR reactions were prepared, consisting of 5.0 µL of bisulfite-converted DNA, 12.5 µL OneTaq 2X Master Mix with Standard Buffer (New England BioLabs, Ipswich, MA, USA), 1.0 µL 10X forward primer and 1.0 µL 10X reverse primer (Integrated DNA Technologies, Coralville, IA, USA), and 5.5 µL NFW. The same reagent volumes were used for the subsequent concentration dependency experiment, but OneTaq Hot Start was used in place of standard OneTaq. In the quantification accuracy experiments, OneTaq Hot Start was again used. Additionally, the template DNA volume for PCR was reduced from 5 µL to 4 µL to preserve the sample, and was compensated for with an additional microliter of NFW. PCR conditions for all three experiments were as follows: 95 °C for 10 min, (95 °C for 30 s, 58 °C for 45 s, 72 °C for 60 s) for 37 cycles, 72 °C for 7 min, and a final 4 °C hold.

### 2.3. Restriction Enzyme Digestion

Digestions were conducted with the PCR products from the quantification accuracy experiments (varying methylation percentage). Digests, with total volumes of 25 µL, consisted of 5 µL of PCR products, 2.5 µL 10X rCutSmart Buffer (New England BioLabs, Ipswich, MA, USA), 0.5 µL 10,000 units/mL BstUI (New England BioLabs, Ipswich, MA, USA), and 17 µL NFW. The negative controls were prepared using the same recipe but with NFW in place of the enzyme. Reactions were incubated at 60 °C for 15 min before analysis.

### 2.4. Microfluidic Electrophoretic Analysis

The samples were analyzed using the LabChip GX Touch II platform (PerkinElmer, Waltham, MA, USA) to control robotic motion, fluids, electric fields, and optics. The microfluidic chip used to analyze the samples was pre-loaded with a PDMS-based gel-dye matrix prepared following the DNA High Sensitivity Assay Guide (PerkinElmer) before each experiment. NFW was used as the sample buffer to keep the sample conductivity low for improved fluorescent labeling and consistent electrophoretic mobility. The microchip was placed inside the LabChip platform, and negative pressure was applied to load the sample directly from the well plate onto the chip. Then, the sample was moved toward the detection window using electric fields. As the sample migrated through the microchannels, it was dynamically labeled, and the DNA fragments of varied sizes were electrophoretically separated. For both the PCR optimization and concentration dependency experiments, 10 µL of each PCR product was diluted in 15 µL NFW; however, all 25 µL from the digestion reactions were analyzed to assess the digests from the quantification accuracy experiments. In this study, all samples were analyzed in triplicate. The resulting electropherograms were analyzed using LabChip GX Reviewer software (PerkinElmer) and DNA concentrations were analyzed by averaging the areas under the *RASSF1* peaks for the three sips. In the experiments involving digests, the percent decrease in peak size corresponded to the percent methylation in the sample. A key innovation during the analysis of the electropherograms, as will be described later, was the way in which the peak areas were evaluated for methylation estimation. Significant troubleshooting went into selecting and optimizing the assay conditions that would maximize the signal yielded by the DNA products to further streamline the analytical portion of the proposed method. The sample preparation and analysis optimization, together with the LabChip capabilities, are what ultimately enabled us to bypass the purification and concentration steps. These improvements allowed for a significant decrease in turnaround time, as highlighted in Table 2. All figures related to the electrophoretic analyses were created using GraphPad Prism 9.

## 3. Results

### 3.1. Primer Design and PCR Optimization for the Amplification of Fully Methylated and Fully Unmethylated Bisulfite Treated Samples

Pure M-DNA and UM-DNA samples were treated with bisulfite conversion reagents and PCR-amplified using the process described in the Materials and Methods section to evaluate the efficacy of the primers and PCR conditions. Each primer set tested yielded varying degrees of success (Table S1, Figure S1), as analyzed with our electrophoresis-based microfluidic analytical platform. Based on the corresponding electropherogram of each primer set and the incidence in both tissue and liquid biopsies of the genes on interest, *RASSF1* was selected. In addition to its high incidence, *RASSF1* is an attractive target, as its primers (Table 3 and primer A from Table S1) flank an amplicon with two distinct CGCG sites.

**Figure 2:**
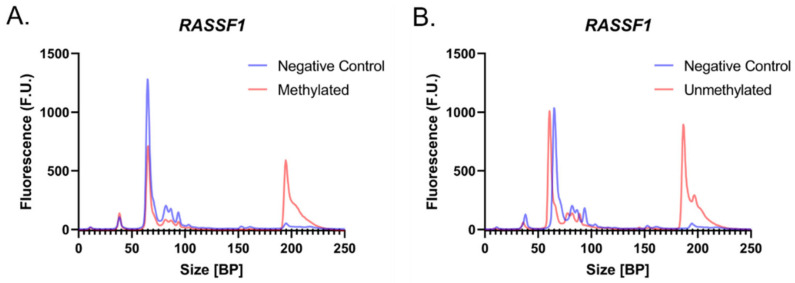
Evaluating primer efficacy. Electropherograms displaying the negative controls (N.C.; blue) and the *RASSF1* region isolated from 100% (**A**) M-DNA (red) and (**B**) UM-DNA (red).

The electropherograms generated with the selected primers display visible peaks corresponding to amplified *RASSF1* regions in 100% M-DNA and 100% UM-DNA, whereas the negative controls did not display peaks at this location (Figure 2A,B), at around 190 bp. While these electropherograms suggest sizes slightly smaller (190 bp) than the expected length of 204 bp, sizing accuracy of this assay is ±10%, which means 190 bp is within the expected region.

### 3.2. Initial Concentration Dependency of the System

**Figure 3:**
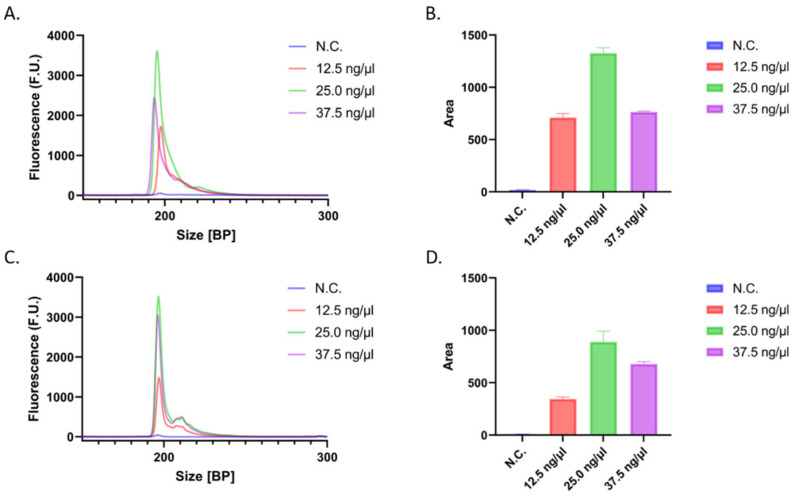
Initial concentration dependency for M-DNA (**A**,**B**) and UM-DNA (**C**,**D**). (**A**) Electropherogram displaying results from input concentrations of 12.5 ng/μL, 25.0 ng/μL, and 37.5 ng/μL of 100% M-DNA. (**B**) Bar graph representing areas under the corresponding curves in the electropherogram from part (**A**). (**C**) Electropherogram displaying results from input concentrations of 12.5 ng/μL, 25.0 ng/μL, and 37.5 ng/μL of 100% UM-DNA. (**D**) Bar graph representing areas under the corresponding curves in the electropherogram from part (**C**).

Since the amount of DNA extracted from clinical samples can differ from patient to patient, we assessed the robustness of our method and potential clinical application with varying input concentrations of DNA. Three 20 μL samples of both M- and UM-DNA with concentrations of 12.5 ng/μL, 25.0 ng/μL, and 37.5 ng/μL were treated with bisulfite conversion reagents and amplified with PCR. While an arbitrary volume of 20 μL was selected during these experiments, if needed, this volume can be scaled down to 5–10 μL, even when analyzing the samples in triplicate. The products were analyzed and the results were summarized by calculating the area under the curves (Figure 3). It was found that the highest product concentration occurred when input DNA, regardless of methylation status, was 25.0 ng/μL. However, while the initial results yield peaks at all three clinically relevant concentrations, it is important to assess whether higher DNA concentrations would affect the accuracy of the system and if dilutions of the samples may be needed. Another noteworthy observation is that the area under the curve for the peak of interest did not appear to follow a linear relationship with concentration. Although additional experiments may be required to determine the exact cause, we believe this may be due to differences in the salt composition of the samples, which would ultimately affect the labeling efficiency of the DNA. However, while this would ideally follow a linear relationship since the proposed assay (Section 3.3) relies on comparing the areas of the same sample digested and non-digested, the labeling efficiency of the two is expected to be the same and should not affect the accuracy of the results.

### 3.3. Methylation Quantification Accuracy

Based on the results from the initial concentration dependency experiments, a set of gradient experiments was conducted in which three samples with an initial DNA concentration of 25.0 ng/μL and varying percentages of methylation (100%, 50%, and 0%) were treated with bisulfite conversion reagents and amplified with PCR. The resulting products were analyzed to verify successful amplification and then treated with BstUI. Corresponding negative controls received NFW rather than enzyme.

**Figure 4:**
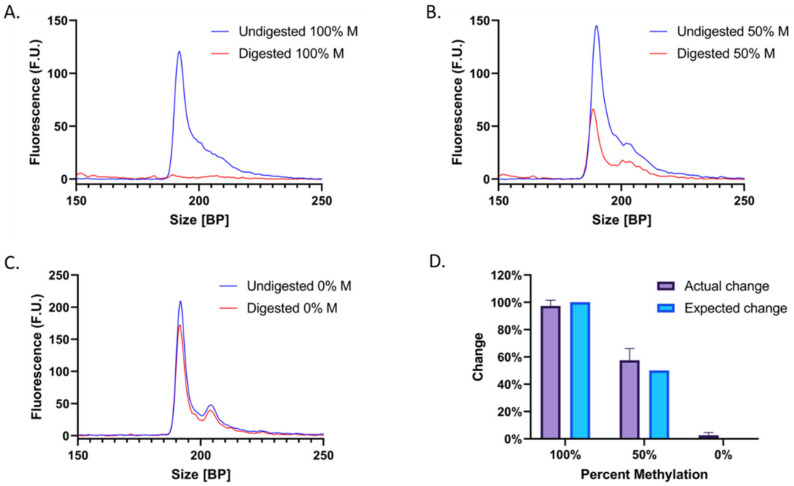
Electropherograms before and after digestion of (**A**) 100% M-DNA, (**B**) 50% M-DNA, and (**C**) 0% M-DNA, with digested samples in red and negative controls (no enzyme) in dark blue. (**D**) Summarized results of the actual change (purple) compared to the expected change (light blue) in concentration after digestion.

As predicted, after digestion with BstUI, the estimated concentration of the *RASSF1* peak decreased proportionately with an increasing percentage of methylation in the sample. Following enzymatic digestion of 100% M-DNA, the *RASSF1* peak decreased by 97.4 ± 4.2% (Figure 4A), compared to an expected decrease of 100% (Figure 4D). This is consistent with the expectation that methylated DNA is not converted during bisulfite treatment and therefore retains its BstUI recognition sequences. The 50% M-DNA (50% UM-DNA) sample peak decreased by 57.6 ± 8.6% (Figure 4B), compared to an expected decrease of 50% (Figure 4D). Lastly, enzymatic digestion of 0% M-DNA (100% UM-DNA) resulted in a peak of approximately equal magnitude to that of the undigested sample, with a decrease of only 2.6 ± 2.0% (Figure 4C), compared to an expected change of 0% (Figure 4D). The general inability of BstUI to digest the unmethylated sample implies a lack of CGCG sequences, indicating the successful conversion of most unmethylated cytosines to thymines. It must be noted that 100% efficiency is generally not possible, and that all three samples displayed an average change that overlapped with the expected changes in peak concentration. Furthermore, experimental results deviated from the expected results by an average of only 4.3%. Here, we show how a methylation-based difference can be translated into a size-based difference for increased separation potential.

Unexpectedly, two adjacent peaks appear at ~200 bp when UM-DNA is analyzed. One potential explanation is that PCR yielded two slightly different products. Amplification of bisulfite-treated UM-DNA poses unique challenges: due to the conversion of all cytosines to thymines, the DNA only contains three distinct bases, which can lead to non-specific primer annealing due to lack of sequence variability. Nonetheless, each of the two adjacent peaks displays a proportional decrease in their area upon digestion (Figure 4B), indicating that both correspond to the *RASSF1* peak and do not interfere with the analysis.

## 4. Discussion

In this study, we sought to devise an epigenetics-based approach for rapid and accessible breast cancer screening. We designed unique primers that isolated a region of a *RASSF1* CpG island containing two distinct sequences of interest, and optimized PCR conditions to obtain high product yields. We proposed several novel modifications to streamline existing CoBRA protocols which, combined with a high-resolution, high-sensitivity system, reduced turnaround time and eliminated the necessity of some steps that required additional reagents and apparatus.

Of the various concentrations of input DNA assessed, 25.0 ng/μL resulted in the highest product yield. Surprisingly, results indicated that the higher concentration may have hindered the amplification and analysis process. However, even 12.5 ng/µL, the lowest concentration of DNA evaluated, was sufficient for analysis. We estimate that *RASSF1* methylation would be quantifiable in a sample with a DNA concentration as low as 9.1 ng/μL, a value obtained by extrapolating the experimental limit of detection of the system (concentration/area), assuming up to 95% methylation, and accounting for sample dilution during digestion. This indicates that our proposed method would be sufficient for detecting methylation in clinical samples containing relatively low concentrations of genomic DNA. Experimental validation of this proposed limit of detection is a necessary next step, along with the evaluation of DNA concentrations exceeding 37.5 ng/µL, the highest value evaluated in this study.

Additionally, the digestion experiments indicated that our system was able to accurately quantify methylation in fully methylated, partially methylated, and fully unmethylated samples, deviating from expected values by only 4.3%. Overall, we obtained promising results that demonstrate the potential of our streamlined system for quantifying methylation in breast cancer samples with high accuracy. To validate the clinical significance of this work, our next steps will involve the use of clinical samples, which will enable us to evaluate the success of our method in the presence of various levels of methylation. This will involve ccfDNA extraction from a blood sample using a standard extraction kit, followed by the workflow outlined in Figure 1 and the Materials and Methods section, as previously shown by other groups [15]. Based on the success in detecting methylation from ccfDNA using the bisulfite treatment, we believe the integration of these samples with our method will be smooth.

Furthermore, the scope of our system is not limited to breast cancer screening. Numerous studies have found that *RASSF1* is a potential biomarker for the diagnosis of lung, cervical, and prostate cancer, implying that our assay and primers could be effective in screening for other diseases [42,43,44]. On the other hand, this indicates that the current specificity of the method would be improved by incorporating additional genes implicated in breast cancer into our assay. Since this is meant to be a screening test before more involved testing, it is important for medical personnel to simultaneously assess the risk profile of the patient to determine whether additional cancer types associated with *RASSF1* should also be considered. Similarly, the use of a multiplex approach could also increase the sensitivity of the screening assay, particularly for diagnostic use, as opposed to simply screening.

Lastly, methylation has also been demonstrated to underly numerous conditions unrelated to cancer, including bipolar disorder, tuberculosis predisposition, hepatocellular carcinoma, and depression [45,46,47,48]. This implies that modification of our current method could yield additional assays that would potentially improve screening and early detection of several different disorders. We hope that this paper underscores the utility of epigenetic modifications in disease screening, and the importance of designing fast, accessible, and accurate methylation-based tests.

## Figures and Tables

**Table 1:** Incidence of gene hypermethylation in breast cancer tumors or tumor cells. Note that, depending on the study, the reported values may be specific to a single site (i.e., NotI site), to a type of tumor (i.e., breast cancer carcinomas), or tumors with a specific gene expression profile (PR + or −), which can significantly affect the incidence percentage and may explain the difference in reported values across studies.

Gene	Incidence (%)	Source(s)
*APC*	10, 49, 51	[13,14,15]
*ARHI*	15–20	[16]
*BRCA1*	5, 13	[13,17]
*CDH1*	53	[14]
*CDKN2A*	31	[18]
*DAPK1*	14	[13]
*E-cadherin*	>50	[19]
*ERα*	25, 79	[20,21]
*FOXA1*	62	[15]
*GSTP1*	21, 30, 40	[13,14,22]
*PR*	46	[20]
*RARβ2*	4, 24, 25	[13,14,23]
*RASSF1*	48, 57, 58, 62, 79, 81	[13,14,15,24,25,26]
*SLIT2*	43	[27]
*SLIT3*	16	[28]
*ROBO1*	19	[29]
*TIMP-3*	5, 27	[13,30]
*TWIST*	48	[14]
*14-3-3σ*	38–96, 91	[31,32]

**Table 2:** Comparison of methods. This table displays differences between the approach described in this paper, CoBRA-GXT, and other methods of quantifying DNA methylation. The number of samples indicated in the automation/high-throughput row excludes the ladder. Please note that the automation/high throughput times include chip priming times when applicable.

	Bisulfite-Sequencing [33]	Standard CoBRA [37]	Bio-CoBRA [38]	Our Method
Analysis method	Sequencing	PAGE gel electrophoresis	Microfluidics (BioAnalyzer)	Microfluidics (LabChip)
Requires sequencing	✓	✕	✕	✕
Requires concentration step	✓	✓	✓	✕
Requires PCR purification step	✓	✓	✓	✕
Number of CpG sites analyzed	Many	One	Not specified	** Two **
Enzyme digestion time	Variable	≥4 h	≥2 h	** 15 min **
Automation/high throughput	Barcoded samples needed, 2 days, low throughput	Manual 10 min/sample (20 samples), medium throughput	Automated 175 s/sample (12 samples), medium throughput	** Automated ** **94 s/sample** **(96 samples), high throughput**

**Table 3:** Information relating to the primers and amplicons associated with the genes of interest.

Gene	Sequence, Fwd. (5′-3′)	Sequence, Rev. (5′-3′)	Expected Amplicon Length (bp)	Annealing Temperature (°C)
*RASSF1*	GGG TTT TAT AGT TTT TGT ATT TAG GTT TTT ATT	CCG CAA CTC AAT AAA CTC AAA CT	204	56

## Data Availability

Not applicable.

## Supplementary Materials

The following supporting information can be downloaded at: https://www.mdpi.com/article/10.3390/biotech12010007/s1, Table S1: Singleplex primer optimization experiments; Figure S1: Representative electropherogram of singleplex experiments.

## References

[1] Siegel R.L., Miller K.D., Fuchs H.E., Jemal A. (2021). Cancer Statistics, 2021. CA Cancer J. Clin..

[2] Pfeiffer R.M., Webb-Vargas Y., Wheeler W., Gail M.H. (2018). Proportion of U.S. Trends in breast cancer incidence attributable to long-term changes in risk factor distributions. Cancer Epidemiol. Biomark. Prev..

[3] Lovelace D.L., McDaniel L.R., Golden D. (2019). Long-Term Effects of Breast Cancer Surgery, Treatment, and Survivor Care. J. Midwifery Womens Health.

[4] Marmot M.G., Altman D.G., Cameron D.A., Dewar J.A., Thompson S.G., Wilcox M. (2013). The benefits and harms of breast cancer screening: An independent review. Br. J. Cancer.

[5] Hortobagyi G.N. (1998). Treatment of Breast Cancer. N. Engl. J. Med..

[6] Bytnar J.A., Byrne C., Olsen C., Witkop C., Martin M.B., Banaag A., Koehlmoos T. (2021). The Impact of Mammography Screening Guideline Changes in a Universally Insured Population. J. Womens Health.

[7] Pace L.E. (2022). False-Positive Results of Mammography Screening in the Era of Digital Breast Tomosynthesis. JAMA Netw. Open.

[8] Tsuruda K.M., Larsen M., Román M., Hofvind S. (2022). Cumulative risk of a false-positive screening result: A retrospective cohort study using empirical data from 10 biennial screening rounds in Breast Screen Norway. Cancer.

[9] Ellisen L.W., Haber D.A. (1998). Hereditary Breast Cancer. Annu. Rev. Med..

[10] Moore L.D., Le T., Fan G. (2012). DNA Methylation and Its Basic Function. Neuropsychopharmacology.

[11] Szyf M. (2012). DNA methylation signatures for breast cancer classification and prognosis. Genome Med..

[12] Cervera R., Ramos A., Lluch A., Climent J. (2016). DNA Methylation in Breast Cancer. Epigenetic Biomarkers and Diagnostics.

[13] Buyru N., Altinisik J., Ozdemir F., Demokan S., Dalay N. (2009). Methylation profiles in breast cancer. Cancer Investig..

[14] Shinozaki M., Hoon D.S., Giuliano A.E., Hansen N.M., Wang H.J., Turner R., Taback B. (2005). Distinct Hypermethylation Profile of Primary Breast Cancer Is Associated with Sentinel Lymph Node Metastasis. Clin. Cancer Res..

[15] Salta S., Nunes S.P., Fontes-Sousa M., Lopes P., Freitas M., Caldas M., Antunes L., Castro F., Antunes P., Palma de Sousa S. (2018). A DNA Methylation-Based Test for Breast Cancer Detection in Circulating Cell-Free DNA. J. Clin. Med..

[16] Yuan J., Luo R.Z., Fujii S., Wang L., Hu W., Andreeff M., Pan Y., Kadota M., Oshimura M., Sahin A.A. (2003). Aberrant Methylation and Silencing of ARHI, an Imprinted Tumor Suppressor Gene in which the Function Is Lost in Breast Cancers. Cancer Res..

[17] Esteller M., Silva J.M., Dominguez G., Bonilla F., Matias-Guiu X., Lerma E., Bussaglia E., Prat J., Harkes I.C., Repasky E.A. (2000). Promoter hypermethylation and BRCA1 inactivation in sporadic breast and ovarian tumors. J. Natl. Cancer Inst..

[18] Herman J.G., Merlo A., Mao L.I., Lapidus R.G., Issa J.P.J., Davidson N.E., Sidransky D., Baylin S.B. (1995). Inactivation of the CDKN2/p16/MTS1 Gene Is Frequently Associated with Aberrant DNA Methylation in All Common Human Cancers. Cancer Res..

[19] Graff J.R., Herman J.G., Lapidus R.G., Chopra H., Xu R., Jarrard D.F., Isaacs W.B., Pitha P.M., Davidson N.E., Baylin S.B. (1995). E-Cadherin Expression Is Silenced by DNA Hypermethylation in Human Breast and Prostate Carcinomas. Cancer Res..

[20] Lapidus R.G., Ferguson A.T., Ottaviano Y.L., Parl F.F., Smith H.S., Weitzman S.A., Baylin S.B., Issa J.P., Davidson N.E. (1996). Methylation of estrogen and progesterone receptor gene 5′ CpG islands correlates with lack of estrogen and progesterone receptor gene expression in breast tumors. Clin. Cancer Res..

[21] Hagrass H.A., Pasha H.F., Ali A.M. (2014). Estrogen receptor alpha (ERα) promoter methylation status in tumor and serum DNA in Egyptian breast cancer patients. Gene.

[22] Esteller M., Corn P.G., Urena J.M., Gabrielson E., Baylin S.B., Herman J.G. (1998). Inactivation of Glutathione S-Transferase P1 Gene by Promoter Hypermethylation in Human Neoplasia. Cancer Res..

[23] Sirchia S.M., Ferguson A.T., Sironi E., Subramanyan S., Orlandi R., Sukumar S., Sacchi N. (2000). Evidence of epigenetic changes affecting the chromatin state of the retinoic acid receptor β2 promoter in breast cancer cells. Oncogene.

[24] Kioulafa M., Kaklamanis L., Mavroudis D., Georgoulias V., Lianidou E.S. (2009). Prognostic significance of RASSF1A promoter methylation in operable breast cancer. Clin. Biochem..

[25] Dammann R., Yang G., Pfeifer G.P. (2001). Hypermethylation of the CpG Island of Ras Association Domain Family 1A (RASSF1A), a Putative Tumor Suppressor Gene from the 3p21.3 Locus, Occurs in a Large Percentage of Human Breast Cancers. Cancer Res..

[26] Feng W., Shen L., Wen S., Rosen D.G., Jelinek J., Hu X., Huan S., Huang M., Liu J., Sahin A.A. (2007). Correlation between CpG methylation profiles and hormone receptor status in breast cancers. Breast Cancer Res..

[27] Dallol A., Da Silva N.F., Viacava P., Minna J.D., Bieche I., Maher E.R., Latif F. (2002). SLIT2, a Human Homologue of the Drosophila Slit2 Gene, Has Tumor Suppressor Activity and Is Frequently Inactivated in Lung and Breast Cancers. Cancer Res..

[28] Dickinson R.E., Dallol A., Bieche I., Krex D., Morton D., Maher E.R., Latif F. (2004). Epigenetic inactivation of SLIT3 and SLIT1 genes in human cancers. Br. J. Cancer.

[29] Dallol A., Forgacs E., Martinez A., Sekido Y., Walker R., Kishida T., Rabbitts P., Maher E.R., Minna J.D., Latif F. (2002). Tumour specific promoter region methylation of the human homologue of the Drosophila Roundabout gene DUTT1 (ROBO1) in human cancers. Oncogene.

[30] Bachman K.E., Herman J.G., Corn P.G., Merlo A., Costello J.F., Cavenee W.K., Baylin S.B., Graff J.R. (1999). Methylation-associated Silencing of the Tissue Inhibitor of Metalloproteinase-3 Gene Suggests a Suppressor Role in Kidney, Brain, and Other Human Cancers. Cancer Res..

[31] Umbricht C.B., Evron E., Gabrielson E., Ferguson A., Marks J., Sukumar S. (2001). Hypermethylation of 14-3-3 σ (stratifin) is an early event in breast cancer. Oncogene.

[32] AFerguson A.T., Evron E., Umbricht C.B., Pandita T.K., Chan T.A., Hermeking H., Marks J.R., Lambers A.R., Futreal P.A., Stampfer M.R. (2000). High frequency of hypermethylation at the 14-3-3 σ locus leads to gene silencing in breast cancer. Proc. Natl. Acad. Sci. USA.

[33] Kurdyukov S., Bullock M. (2016). DNA Methylation Analysis: Choosing the Right Method. Biology.

[34] Phillips K.A., Pletcher M.J., Ladabaum U. (2015). Is the $1000 Genome really $1000? Understanding the full benefits and costs of genomic sequencing. Technol. Health Care.

[35] Pruneri G., De Braud F., Sapino A., Aglietta M., Vecchione A., Giusti R., Marchiò C., Scarpino S., Baggi A., Bonetti G. (2021). Next-Generation Sequencing in Clinical Practice: Is It a Cost-Saving Alternative to a Single-Gene Testing Approach?. Pharm.-Open.

[36] MedlinePlus (2020). What Is the Cost of Genetic Testing, and How Long Does It Take to Get the Results?. https://medlineplus.gov/.

[37] Xiong Z., Laird P.W. (1997). COBRA: A sensitive and quantitative DNA methylation assay. Nucleic Acids Res..

[38] Brena R.M., Auer H., Kornacker K., Plass C. (2006). Quantification of DNA methylation in electrofluidics chips (Bio-COBRA). Nat. Protoc..

[39] Howe K.L., Achuthan P., Allen J., Allen J., Alvarez-Jarreta J., Amode M.R., Armean I.M., Azov A.G., Bennett R., Bhai J. (2021). Ensembl 2021. Nucleic Acids Res..

[40] Robinson J.T., Thorvaldsdóttir H., Winckler W., Guttman M., Lander E.S., Getz G., Mesirov J.P. (2011). Integrative genomics viewer. Nat. Biotechnol..

[41] Gardiner-Garden M., Frommer M. (1987). CpG Islands in vertebrate genomes. J. Mol. Biol..

[42] Walter R.F.H., Rozynek P., Casjens S., Werner R., Mairinger F.D., Speel E.J.M., Zur Hausen A., Meier S., Wohlschlaeger J., Theegarten D. (2018). Methylation of L1RE1, RARB, and RASSF1 function as possible biomarkers for the differential diagnosis of lung cancer. PLoS ONE.

[43] Cohen Y., Singer G., Lavie O., Dong M., Beller U., Sidransky D. (2003). The RASSF1A Tumor Suppressor Gene Is Commonly Inactivated in Adenocarcinoma of the Uterine Cervix. Clin. Cancer Res..

[44] Daniunaite K., Jarmalaite S., Kalinauskaite N., Petroska D., Laurinavicius A., Lazutka J.R., Jankevicius F. (2014). Prognostic Value of RASSF1 Promoter Methylation in Prostate Cancer. J. Urol..

[45] Kaminsky Z., Tochigi M., Jia P., Pal M., Mill J., Kwan A., Ioshikhes I., Vincent J.B., Kennedy J.L., Strauss J. (2012). A multi-tissue analysis identifies HLA complex group 9 gene methylation differences in bipolar disorder. Mol. Psychiatry.

[46] Andraos C., Koorsen G., Knight J.C., Bornman L. (2011). Vitamin D receptor gene methylation is associated with ethnicity, tuberculosis, and Taq I polymorphism. HIM.

[47] Li B., Huang H., Huang R., Zhang W., Zhou G., Wu Z., Lv C., Han X., Jiang L., Li Y. (2020). SEPT9 Gene Methylation as a Noninvasive Marker for Hepatocellular Carcinoma. Dis. Markers.

[48] Chen D., Meng L., Pei F., Zheng Y., Leng J. (2017). A review of DNA methylation in depression. J. Clin. Neurosci..

