# Supplementary material for: An Optimized CoBRA Method for the Microfluidic Electrophoresis Detection of Breast Cancer Associated RASSF1 Methylation
Source: BioTech (Basel). 2023 Jan 9;12(1):7. doi: 10.3390/biotech12010007 (PMC9844460; doi:10.3390/biotech12010007)

Suppplementary Material: An optimized CoBRA-LabChip method for the microfluidic detection of breast cancer-associated DNA methylation

Table S1. Singleplex primer optimization experiments. Results of using different primer sequences to amplify target regions of various breast cancer-implicated genes.

Primer	Gene	Sequence, Fwd. (5'-3')	Sequence, Rev. (5'-3')	Expected amplicon length (bp)	Annealing temperature (°C)	Results
**A**	*RASSF1*	GGG TTT TAT AGT TTT TGT ATT TAG GTT TTT ATT	CCG CAA CTC AAT AAA CTC AAA CT	204	56	Successful amplification
**B**	*RASSF1*	GGG TTT TAT AGT TTT TGT ATT TAG GTT TTT A	CTC AAT AAA CTC AAA CTC CCC CAA CAT A	198	56	Successful amplification
**C**	*FOXA1*	GGT TTT TGT TTG GTA TTT TTT TTG TAG T	ACC TAC AAC TAA AAA CAA ATA AAT CAC A	371	56	Unsuccessful amplification of target region
**D**	*GSTP1*	TTT TGT TGG GGA TTT GGG AAA	AAA AAA ACA CCC TAA AAT CCC C	81	56	Successful amplification
**E**	*GSTP1*	GGG AAA GAG GGA AAG GTT TTT TT	CAA AAA AAC ACC CTA AAA TCC C	67	56	Successful amplification but lower yield
**F**	*GSTP1*	AAA GAG GGA AAG GTT TTT TTG GTT A	CAA AAA AAC ACC CTA AAA TCC C	64	56	Successful amplification
**G**	*GSTP1*	GGA GGT TGA AGT AGA ATT GTT TGA AT	CCC ACT TCA CAA AAC AAA AAA AA	268	56	Unsuccessful amplification of target region
**H**	*GSTP1*	ATA ATT TTA TTT ATT CGG GAG GTT GAA G	CGC TAT AAC CCA AAC TAA AAT ACA ATA A	109	56	Unsuccessful amplification of target region
**I**	*GSTP1*	TTA TAA TTT TAT TTA TTC GGG AGG TTG A	ACG CTA TAA CCC AAA CTA AAA TAC AAT	112	56	Unsuccessful amplification of target region

Figure S1. Representative electropherogram of singleplex experiments. Electropherogram resulting from using primer set D to amplify (a) methylated (M), and (b) unmethylated (UM) DNA. The electropherograms corresponding to amplified *GSTP1* regions displayed amplification at 85 and 86 bp for the 100% M-DNA (a) and 100% UM-DNA (b), respectively, which falls within the desired region of 81 ± 10%.

(a)

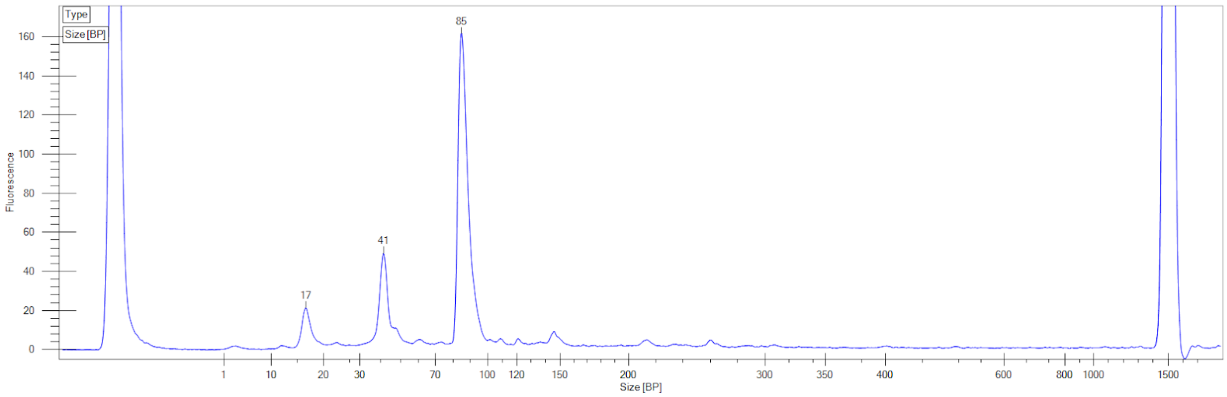

(b)

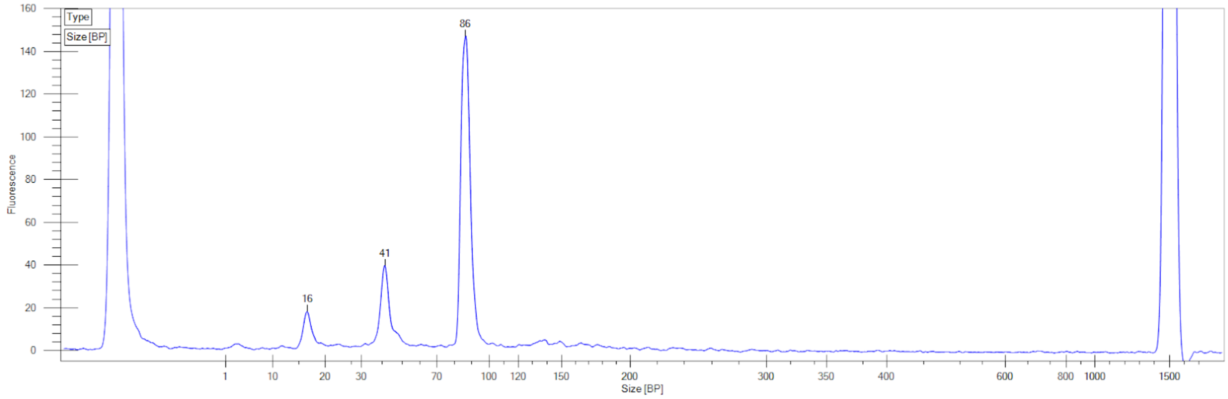

Supplement: Supplementary file 1 [file biotech-12-00007-s001.zip › biotech-2116196-supplementary.pdf]
